# Diagnostic Accuracy of Serum P16ink4A and FOX-P3 Concentrations for Detection of Cervical Lesions Among Women Attending a Cervical Cancer Clinic in Western Uganda: A Case-Control Study

**DOI:** 10.1155/ancp/1931921

**Published:** 2025-05-06

**Authors:** Frank Ssedyabane, Nixon Niyonzima, Joseph Ngonzi, Josephine Nambi Najjuma, Alexcer Namuli, Christopher Okeny, Doreen Nuwashaba, Abraham Birungi, Rogers Kajabwangu, Thomas C. Randall, Cesar M. Castro, Hakho Lee, Deusdedit Tusubira

**Affiliations:** ^1^Department of Medical Laboratory Science, Faculty of Medicine, Mbarara University of Science of Science and Technology, Mbarara, Uganda; ^2^Research and Training Directorate, Uganda Cancer Institute, Kampala, Uganda; ^3^Department of Obstetrics and Gynecology, Mbarara University of Science of Science and Technology, Mbarara, Uganda; ^4^Department of Nursing, Mbarara University of Science of Science and Technology, Mbarara, Uganda; ^5^Department of Pathology, Mbarara University of Science of Science and Technology, Mbarara, Uganda; ^6^Department of Global Health and Social Medicine, Massachusetts General Hospital, Harvard Medical School, Boston, Massachusetts, USA; ^7^Centre for Systems Biology, Massachusetts General Hospital, Harvard Medical School, Boston 02114, Massachusetts, USA; ^8^Cancer Centre, Massachusetts General Hospital, Harvard Medical School, Boston 02114, Massachusetts, USA; ^9^Department of Radiology, Massachusetts General Hospital, Harvard Medical School, Boston 02114, Massachusetts, USA; ^10^Department of Biochemistry, Mbarara University of Science of Science and Technology, Mbarara, Uganda

**Keywords:** accuracy, cervical cancer, cervical intraepithelial lesions, FOXP3, P16ink4A, serum

## Abstract

**Introduction:** Expression of P16ink4A and FOXP3 is correlated with the grades of cervical lesions. In this study, we determined the diagnostic accuracy of serum P16ink4A and FOXP3 concentrations for detection of cervical intraepithelial neoplasia (CIN) and cervical cancer (CC) in a rural setting in Southwestern Uganda.

**Material and Methods:** CIN and CC cases (93 each before treatment), and 93 controls were identified. Clinical and demographic data were documented before quantifying serum P16ink4A and FOXP3 concentrations using quantitative ELISA kits. Cases were confirmed by cytology and/or histology. We employed descriptive statistics, cross-tabulation, and receiver operating curves (ROC) using statistical software for data science (STATA) 17. *p*-values <0.05 were considered statistically significant.

**Results:** Serum FOXP3 concentration of 0.0545 ng/mL < showed moderate sensitivity (32.22% and 57.78%) for detection of CIN and CC from healthy controls, respectively. It also showed a moderately high specificity of 68.89% for detection of both CIN and CC from healthy controls (AUC-0.6014 and 0.7679, respectively). Serum P16ink4A concentration of 0.946 ng/mL < showed moderate sensitivities (50.00% and 60.00%) and specificities (56.67% and 55.56%) for the detection of CIN and CC from healthy controls, respectively (AUC-0.6085 and 0.7592, respectively). A combination of elevated serum FOXP3 and P16ink4A showed very low sensitivities of 18.89% in detecting CIN from healthy controls and 33.33% for detecting CC from healthy controls. This combination showed high specificity of 83.33% in detecting both CIN and CC from healthy controls (AUC-0.5992 and 0.7642, respectively).

**Conclusion:** Although serum P16ink4A and FOXP3 concentrations showed moderate accuracy, their combination was more specific than sensitive. This combination has a high potential to be applied for diagnosis rather than screening for cervical lesions, at least in the Ugandan population. Combinations of P16ink4A and FOXP3 with other biomarkers could improve diagnostic accuracies. Additionally, studies could be conducted to assess the performance of these biomarkers in the detection of cervical lesions in specific populations, say Human Immunodeficiency Virus (HIV)-positive and HIV-negative populations.

## 1. Introduction

Challenges with current screening and diagnostic techniques contribute to the slow progress toward cervical cancer (CC) eradication, particularly in low-resource environments. Such challenges have been exacerbated by low uptake [1, 2], leading to minimal impact on CC mortality and morbidity. Traditional tests such as visual inspection with acetic acid (VIA) and the Pap test are discomforting for patients during specimen collection and also carry along many barriers towards uptake [3–8]. Both tests yield subjective results and thus bear challenges with respect to uniformity [9]. The Pap test has been reported to have low to moderate sensitivity [10] when screening for cervical neoplasms, and leads to many repeat tests and many unnecessary colposcopies [11].

Usually, an abnormal Pap test is relied on, and more invasive and expensive investigations are pursued to confirm the presence or absence of cancerous or precancerous lesions [12]. These investigations include Human Papilloma Virus (HPV) DNA, colposcopy, histology and/or immunohistochemistry to diagnose CC or precancers. Histological examination of cervical biopsies can reveal precancerous lesions, cervical squamous cell carcinoma or cervical adenocarcinoma. However, histological investigations on cervical preparations are also not only discomforting and invasive during specimen collection but also yield subjective results [9, 13]. There is significant inter and intraobserver variability as well as poor adherence to standards [9, 14]. Colposcopy is equally invasive and delays treatment options, resulting in extra costs and risks to patients [12].

Screening for cancer of the cervix with HPV DNA tests has been shown to be more sensitive compared to cytology [15]. However, its major shortfall is the low positive predictive value, especially for high-grade lesions [15]. Several biomarkers have been reported to be potentially useful in the screening and diagnosis of cervical lesions. Studies have suggested that some biomarkers are simpler to detect, noninvasive during specimen collection and can detect cervical lesions with high sensitivity and specificity [16, 17]. Some of these biomarkers have been used in immunohistochemical (IHC) studies on tissue sections. However, immunohistochemistry also has its general shortcomings, such as false positives and poor accuracy for low-grade tumours [18]. This calls for more sensitive and specific biomarkers to better identify dysplastic cells [8].

Biomarkers, including cyclin-dependent kinase inhibitor 2A (P16ink4A) and forkhead box p3 (FOXP3), have been successfully applied in IHC techniques. IHC expression of P16ink4A is known to be related to the degree of histological dysplasia and malignancy [19]. Relatedly, overexpression of P16ink4A is not only associated with disease-free survival but also increased overall survival, thus a better prognostic marker [20]. In some instances, P16ink4A IHC studies have been carried out in combination with KI-67 [21–23]. This duo staining is now used as an adjunctive test in screening for CC [24].

P16ink4A is a cell cycle regulator protein whose amount and role are tightly regulated in normal cells [25, 26]. It is a tumour suppressor protein that inhibits cyclin-dependent kinases 4 and 6 by phosphorylating the retinoblastoma (Rb) protein [26]. During a transforming HPV infection, the viral oncogene E7 binds the Rb protein, which results in increased levels of P16ink4A [27]. It is from such a background that P16ink4A is believed to serve as a surrogate marker for persistent HR HPV infection [28, 29]. In fact, P16ink4A has been described as a sensitive and specific marker for dysplastic cervical cells and thus useful for CC screening and diagnosis [30–34].

Zhang et al. [35] stated that the sensitivity of CC screening tests, like HPV DNA and Pap smear cytology, may be underestimated when compared with an imperfect gold standard, histology. They revealed that P16ink4A IHC has an impact on determining the performance of CC screening tests [35]. Moreover, there is improved interobserver agreement of CIN2+ with the conjunctive use of histology with P16ink4A IHC compared with histology alone [36].

Forkhead/winged-helix transcription factor box P3 (FOXP3) is a regulator for regulatory T cells (Treg) development and function, and belongs to the forkhead protein family of transcription regulators [37]. FOXP3 is said to accelerate cancer development through a number of mechanisms including increasing the rate of lymphangiogenesis [38]. Also, T cells, on which FOXP3 is expressed, have the potential to block the immune response, which itself leads to development of malignancy [39–42]. Earlier studies by Karanikas et al. [43], Ma et al. [44], Tan et al. [45], Fu et al. [46], Ladoire et al. [47] and Luo et al. [37] revealed that FOXP3 is expressed both in the cytoplasm and nucleus of cancer cells. Other IHC studies by Luo et al. [37] showed that there is no demonstrable FOXP3 expression in normal cervical tissue, yet there is a significant increase in FOXP3 expression from CIN1 all through to CC. This confirms earlier observations that FOXP3 expression is associated with development, progression and prognosis of cancers [38, 43–47]. It is also worth noting that FOXP3 expression is not only significantly and positively related to P16ink4A expression [37] but also both biomarkers are associated with cervical lesions, as shown by studies in a Ugandan population [48, 49]. A positive correlation also exists between the expression of FOXP3 and VEGF-C and CC lymphangiogenesis [38].

Nonetheless, they can be objectively quantified and measured in blood using methods that can be standardised [50–54]. The analytical performance and diagnostic utility of these biomarkers for detecting cervical neoplasms can thus be rigorously assessed in low-resource settings such as Uganda. This study aimed to describe the diagnostic accuracy of serum P16ink4A and FOXP3 concentrations for the detection of cervical intraepithelial lesions among women attending the CC clinic of Mbarara Regional Referral Hospital (MRRH), Southwestern Uganda.

## 2. Methods

### 2.1. Study Design and Setting

This unmatched case–control diagnostic accuracy study included all patients who sought CC care at the CC clinic of MRRH. Women cytologically or histologically confirmed to have cervical lesions (cervical intraepithelial neoplasia [CIN] or CC) were regarded as cases. Women who were negative for intraepithelial lesions or malignancy were regarded as controls. The outcomes of interest were cervical intraepithelial lesions, while the exposures were serum concentrations of P16ink4A and FOXP3. MRRH is a regional referral hospital in rural Southwestern Uganda, whose catchment area is estimated to have a population of four million people [55]. This hospital also serves neighbouring countries, including Tanzania, Burundi, Rwanda and the Democratic Republic of Congo. The clinic runs 5 days a week, and its annual attendance is estimated to be 4000 women. At the clinic, routinely offered screening tests include VIA, colposcopy, pap smear cytology, and most recently, HPV DNA. Those who test positive for the screening test are subjected to histopathological evaluation. Women confirmed to have cervical premalignant lesions receive cryotherapy or thermocoagulation treatment modalities. Those confirmed to have CC undergo gynaecologic cancer surgery or are referred to the Uganda Cancer Institute for radiotherapy and chemotherapy, depending on clinical stage.

### 2.2. Participants

**Figure 1:**
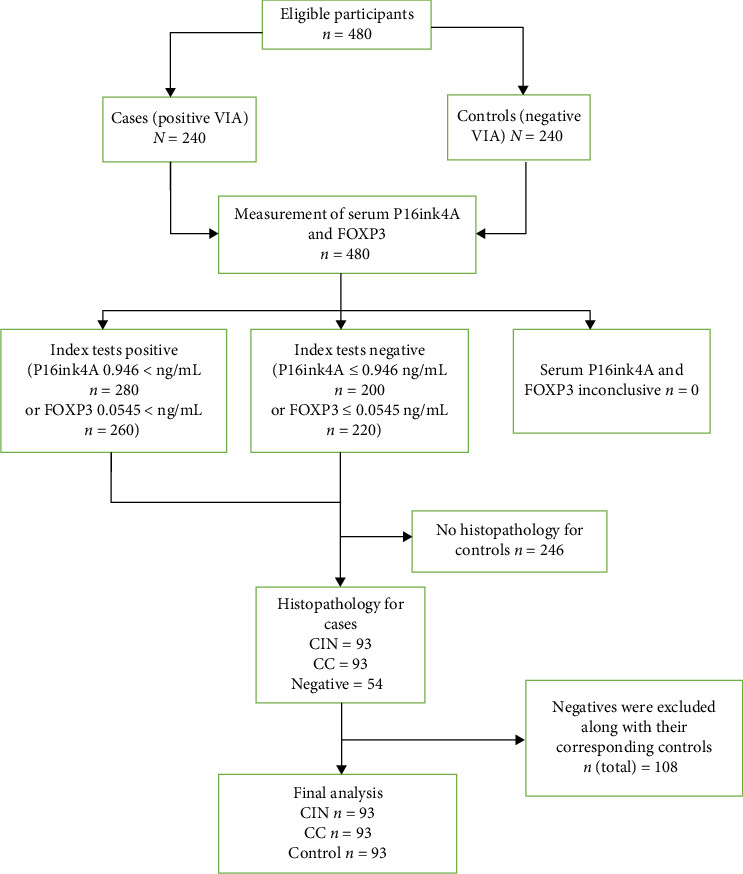
STARD flow diagram of participants through enrolment, eligibility and outcomes of the study. CC, cervical cancer; CIN, cervical intraepithelial neoplasia; VIA, visual inspection with acetic acid.

We recruited 480 participants for this study and included 279 participants in the final analysis, as shown in Figure 1. These included 93 cases with CIN, 93 controls with CC cases and 93 unmatched controls from among women seeking CC services at the CC clinic of MRRH. The recruitment used the incidence density sampling method to obtain controls. Every time we recruited a case, a control was identified until we reached the required sample size from April 2022 to January 2024.

### 2.3. Inclusion and Exclusion Criteria

This study included all women of 21 years and above receiving CC care at the MRRH CC clinic; provided written informed consent to participate in the study, and later confirmed either positive or negative for cervical lesions (CIN or CC). All those women who were moribund and those who were undergoing treatment for already diagnosed cervical lesions were excluded from the study.

### 2.4. Data Collection

#### 2.4.1. Demographic Data Collection

After provision of routine and standard care, and acquisition of written informed consent, study participants were helped by a trained research assistant to collect demographic data using a validated questionnaire. The data collected included age, residence, highest level of education, marital status, as well as clinical information, such as Human Immunodeficiency Virus (HIV) status, usage of family planning, family planning method, history of high blood pressure and history of diabetes.

#### 2.4.2. Blood Collection, Measurement and Interpretation of Serum P16ink4A and FOX-P3 Concentrations

Four millilitres of nonfasting venous blood were drawn aseptically from every study participant by venipuncture from the mid-cubital vein and dispensed into plain vacutainers. Specimens were labelled using unique identification codes, allowed to clot and later centrifuged for 15 min at 1000 × *g* at 2–8°C. The serum was then transferred into cryovial tubes and stored at −80°C until run in batches.

Quantitative measurement of P16ink4A concentration was done using Human CDKN2A (cyclin dependent kinase inhibitor 2A) ELISA Kit, Elabscience Biotechnology Inc. This kit is designed with a sensitivity of 0.38 ng/mL and a detection range of 0.63–40 ng/mL. Actual measurements using this ELISA kit were based on the sandwich-ELISA principle, following manufacturer's instructions.

Quantitative measurement of FOXP3 concentration was done using Human FOXP3 (forkhead/winged-helix transcription factor box P3) ELISA Kit, Elabscience Biotechnology Inc. This kit is designed with a sensitivity of 0.19 ng/mL and a detection range of 0.31–20 ng/mL. Actual measurements using this ELISA kit were also based on the sandwich-ELISA principle, following manufacturer's instructions.

The optical density (OD) for each biomarker was measured using a microplate reader set at a wavelength of 450 nm ± 2 nm. The OD value was proportional to the concentration of human CDKN2A or FOXP3. For P16ink4A, we used a dilution of 1:3, and this was considered all through the final calculations for concentrations. All samples were analysed together with reference standards. For P16ink4A, we created two categories of concentrations. These included low P16ink4A concentration (≤0.946 ng/mL) and raised concentration (0.946 < ng/mL) as determined and used previously [48]. For FOXP3, we also created two categories of concentrations calculated using the cut point by Phil Clayton, which determines a cut-off point (0.0545) for FOXP3 on the ROC curve that is closest to the point with perfect diagnostic values for sensitivity and specificity. The first category was named “low FOXP3 concentration” (≤0.0545 ng/mL), while the second category was named “raised concentration” (0.0545 < ng/mL).

#### 2.4.3. Reference Standard

For cervical cytological specimens, a sterile speculum was used to provide clear access to the cervix, and samples from the endo and ectocervix were collected and transferred to a clean, well-labelled glass slide. The smears were immediately fixed by immersing them in 95% ethyl alcohol for 15 min and stained using the Papanicolaou staining technique as used previously [56]. The specimens were examined and then reported following the Bethesda system 2014 [57].

For women eligible for biopsy, a cone/punch biopsy sample was obtained for histological examination. Biopsies were fixed for 48 h in 10% formalin and then processed using procedures described by Dey [56] in an open system tissue processor for 14 h using the Slee tissue processor. The tissues were embedded in paraffin wax and sections cut at 3–5 micrometres (μm), and stained with the haematoxylin and eosin (H&E) staining procedure [56]. The outcomes of biopsies were reported as CIN (CIN1, CIN2, CIN3/carcinoma in situ), or invasive CC.

Clinical information about study participants was availed to pathologists who read the cytological and histological preparations from study participants, since it is a standard requirement. However, results from the index test were not provided to the examiners of cytology and histological specimens and vice versa.

#### 2.4.4. Data Management and Analysis

Data were entered into an Excel spreadsheet (Microsoft Office Professional Plus 2013, version 15.0.4675.1003, Microsoft Inc. USA) and imported into statistical software for data science (STATA) 17 (StataCorp LLC, College Station, Texas, United States) software, using password protected computers. The study population was described using frequencies, means ± standard deviations (SDs), or median values for continuous variables as well as frequencies and proportions for categorical variables.

The continuous variables between the groups were compared by *t*-test or ANOVA, whereas the categorical variables were compared by Chi-square or Fisher's exact tests. Sensitivity and specificity of serum P16ink4A, FOXP3 or their combination for the diagnosis of CIN or CC from healthy controls were achieved by use of 2 × 2 tables (cross-tabulation) generated from the results obtained in the study. Sensitivity and specificity were reported as proportions with 95% confidence intervals (CIs). The receiver operating characteristic (ROC) area under the curve (AUC) for P16ink4A, FOXP3 or their combination for the diagnosis of CIN or CC from healthy controls were reported as a proportion with 95% CIs. The results presented in the form of tables, graphs and images/micrographs were appropriate. *p*-values of <0.05 were considered statistically significant.

## 3. Results

### 3.1. Population Characteristics

A total of 279 participants were included in the analysis: 93 were positive for CC, 93 were positive for CIN and 93 were negative for any cervical lesion (controls). The mean age of CC cases was significantly (*p* < 0.001) higher (51.1 ± 13.1) than CIN cases (35.1 ± 7.8) and 38.6 ± 8.7 in controls. The majority of participants, 23% (21/93) of CC cases, 33% (31/93) of CIN cases, and 43% (40/93) of controls, belonged to the 40–49 age bracket. This difference in distribution was also statistically significant (*p* < 0.001). More than half of the study participants, 57% (53/93) of CC cases, 61% (57/93) of CIN cases and 55% (51/93) of controls, were married (*p* < 0.001). The highest level of education among the study participants was generally primary level and below, with 55% (51/93) of CC cases having attained a maximum of preprimary, with 52% (48/93) CIN cases and 45% (42/93) controls reporting to have attained a maximum of primary school education. HIV positivity was reported in 76% (71/93) CC cases, 54% (50/93) of CIN cases and 44% (41/93) of controls and this difference in distribution was statistically significant (*p* < 0.001).

The most common presenting complaint among study participants was cervicitis, with a proportion of 59% (55/93) among CC cases, 72% (67/93) among CIN cases, and 67% (62/90) among controls, and this difference in distribution was also statistically significant (*p* < 0.001) as shown in Table 1.

### 3.2. Individual and Combined Diagnostic Accuracy of Serum P16ink4A and FOXP3 Concentrations for the Detection of CIN and CC From Healthy Controls

A raised serum FOXP3 concentration (above 0.0545 ng/mL) showed moderate sensitivity of 32.22% and 57.78% for the detection of CIN and CC from healthy controls, respectively. It also showed a moderately high specificity of 68.89% for detection of both CIN and CC from healthy controls.

Raised serum P16ink4A concentration (above 0.946 ng/mL) showed moderate sensitivities (50.00% and 60.00%) and specificities (56.67% and 55.56%) for the detection of CIN and CC from healthy controls, respectively.

However, a combination of raised serum FOXP3 and P16ink4A showed very low sensitivities of 18.89% in detecting CIN from healthy controls and 33.33% for detecting CC from healthy controls. This combination showed high specificity of 83.33% in detecting both CIN and CC from healthy controls, as shown in Table 2.

**Figure 2:**
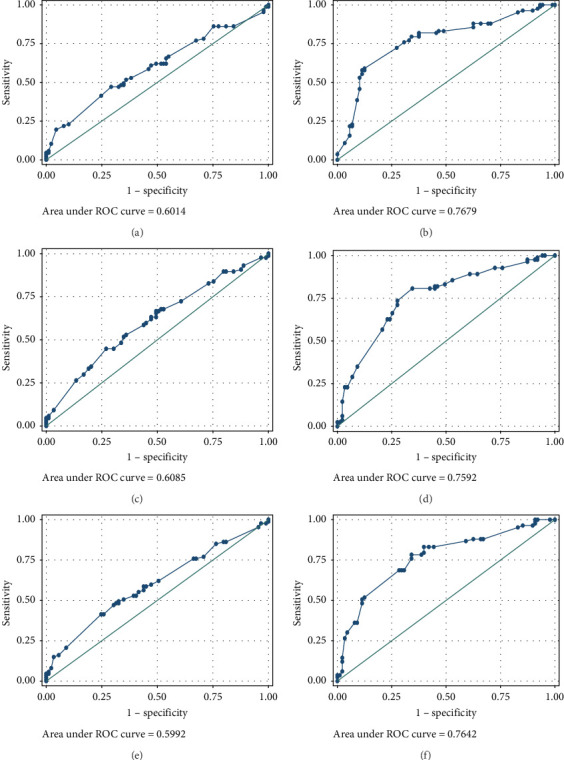
ROC showing diagnostic accuracy of FOXP3 in the detection of CIN from healthy controls (A) and detection of CC from healthy controls (B). The diagnostic accuracy of P16ink4A in the detection of CIN from healthy controls (C) and detection of CC from healthy controls (D). The combination of the two biomarkers for the detection of CIN (E) and CC from healthy controls (F).

The ROC analyses displayed an AUC of 0.6014 for raised serum FOXP3 in the detection of CIN from healthy controls (Figure 2A), and an AUC of 0.7679 for raised serum FOXP3 in the detection of CC from healthy controls (Figure 2B). The ROC also showed an AUC of 0.6085 for raised serum P16ink4A to detect CIN from healthy controls (Figure 2C) and an AUC of 0.7592 for raised serum P16ink4A to detect CC from healthy controls (Figure 2D). The combination of the two biomarkers showed an AUC of 0.5992 for the detection of CIN (Figure 2E) and 0.7642 for the detection of CC from healthy controls (Figure 2F).

## 4. Discussion

This study provides evidence of the possible usage of blood-based biomarkers for the diagnosis of cervical lesions. We present the diagnostic utility of serum Forkhead/winged helix transcription factor box P3 (FOXP3) and P16ink4A as biomarkers for cervical precancerous and or cancerous lesions.

FOXP3 is a member of the forkhead transcription factor family, which is mainly expressed in a subset of CD4+ T-cells that play a suppressive role in the immune system. It suppresses the function of nuclear factor of activated T cells (NFAT) and nuclear factor-kappa B (NF-kB) and subsequently the expression of many genes, including IL-2 and effector T-cell cytokines [58].

In this study, raised FOXP3 serum concentration showed low sensitivity (32.22%) for the detection of CIN, and a moderate sensitivity (57.78%) for the detection of CC. We also observed a moderately high specificity (68.89%) for the detection of both CIN and CC.

The low sensitivity and specificity observed in our study render serum FOXP3 concentration as a test with low value as a screening as well as a diagnostic tool in our population. The ROC for raised serum FOXP3 in our population showed an AUC of 0.6014 for the detection of CIN and 0.7679 for CC. This further demonstrated its limitations in the detection of precancerous and cancerous cervical lesions in our study population. This may be attributed to the demography of our study population.

It is worth noting that 70% of our study participants with CC were 40 years and above. FOXP3 expression diminishes with advancing age, and hence in the elderly, the proportion of resting T-reg drops, while activated T-reg and FOXP3^low^ expressing T cells increases [59]. Our findings were not in agreement with Xu et al. [60], who postulated that circulating autoantibody to FOXP3 reflects continuous development of the cervical lesion, and this may be a potential biomarker with early prognostic values for CC.

We observed that almost half of our population was HIV positive, who would be expected to have reduced numbers of T cells and, therefore, reduced expression of FOXP3 [61–64]. However, this study population had earlier been described to be immunocompetent, as indicated by the CD4 counts and HIV viral load [48, 49].

Raised serum P16ink4A concentration has shown potential to be an effective CC screening test in low-resource settings based on its molecular function and performance on IHC and in prior studies in general populations [65]. From our study, raised P16ink4A serum concentration had moderate sensitivity (50.00% and 60.00%) and specificity (56.67% and 55.56%) for the detection of CIN and CC. This demonstrates its low diagnostic utility in the screening and diagnosis of cervical precancerous or cancerous lesions. Our findings underscore earlier reports from IHC studies, which presented P16ink4A as a potential biomarker of high-grade CIN and invasive CC [66]. Several other studies on cervical specimens agree with our finding. For instance, Wu et al. [65] reported that P16ink4A ELISA on cervical samples did not perform well as a screening tool, with only 30.8% cases of correct P16ink4A prediction to detect or rule out CIN2+, and 69.2% cases of incorrect P16ink4A prediction. They also mentioned that P16ink4A was a promising biomarker in measuring progression of cervical disease, but also cited difficulties in choosing a numerical threshold to distinguish CIN1 from CIN2+ in a population of women with high rates of HPV positivity and low-grade dysplasia [65]. Earlier reports by Balasubramanian et al. [67] disagreed with our study. They reported that P16ink4A enhanced ELISA had a sensitivity of 91.8% and specificity of 86.0%, demonstrating its diagnostic utility in the screening for cervical lesions [67]. This could however be attributed to the difference in sample types as they employed liquid-based cytology samples. Leung et al. [68] also demonstrated the feasibility of quantifying P16ink4A by ELISA in fresh cervical samples, and its potential as an adjunct to existing screening strategies in the identification of high-grade dysplastic lesions using liquid-based cytology.

A combination of FOXP3 and P16ink4A displayed very low sensitivity of 18.89% in detecting CIN and 33.33% for CC, but rather high specificity of 83.33% in detecting both CIN and CC among our study participants. This presents their combined effects as a relatively good diagnostic tool but a poor screening tool for cervical lesions.

We earlier reported that the cost of HPV DNA testing is average, 20 dollars in Uganda [48]. P16ink4A and FOXP3 serum quantification presents an opportunity for a cheaper alternative, since their ELISA tests can go for as low as 8 dollars. This low cost shows the high likelihood of improving CC screening uptake in low-resource settings and eventually reducing the CC burden.

This study might have suffered from misclassification bias, especially during the selection and allocation of controls. Therefore, there might have been some participants who were actually not meant to be controls, and this might have influenced statistical inferences. We did not calculate predictive values as the nature of the study design does not allow. The prevalence/incidence, as seen in the column sum in the 2 × 2 table of participants classified as positive and negative with the reference test, is an artificial result of the study design. Also, we never stratified our analyses based on grades of lesions, whether CIN or CC and hence could not provide accuracy statistics for each grade. This is a missed opportunity, as there could have been some significant observations of sensitivity or specificity for some grades of cervical lesions. This being an unmatched case-control study, we also did not evaluate the specific performance of these biomarkers in HIV-negative or HIV-positive participants. This warrants further investigation.

This study was designed with adequate statistical power to support statistical conclusions. We also used standard methods for confirmation of cases, which helped minimise classification bias for controls.

## 5. Conclusion

Serum P16ink4A and FOXP3 concentrations showed moderate accuracy for the detection of CIN and CC from healthy controls. Their combination was more specific than sensitive and better applicable for diagnosis than screening for cervical lesions. Combinations of P16ink4A and FOXP3 with other biomarkers could improve diagnostic accuracy. Additional studies could be conducted to assess the performance of these biomarkers in the detection of cervical lesions in specific populations, say HIV-positive and HIV-negative populations.

## Figures and Tables

**Table 1:** Demographic characteristics of participants.

Variable	Category	Controls	Cases		Test	*p*-Value
		*N* = 93	CIN	CC		
			*N* = 93	*N* = 93		
Age	—	38.6 (8.7)	35.1 (7.8)	51.1 (13.1)	ANOVA	<0.001

Age group	21–29	19 (20%)	23 (25%)	2 (2%)	Fisher's exact	<0.001
	30–39	27 (29%)	38 (41%)	20 (22%)		
	40–49	40 (43%)	31 (33%)	21 (23%)		
	50–59	7 (8%)	1 (1%)	20 (22%)		
	60-max	0 (0%)	0 (0%)	30 (32%)		

Region	Central	2 (2%)	2 (2%)	6 (6%)	Fisher's exact	<0.001
	Other districts	45 (48%)	53 (57%)	71(76%)		
	Mbarara	46 (49%)	38 (41%)	16 (17%)		

History of high BP	No	73 (78%)	70 (75%)	73 (78%)	Chi-square	0.82
	Yes	20 (22%)	23 (25%)	20 (22%)		

History of diabetes	No	77 (83%)	81 (87%)	72 (77%)	Chi-square	0.28
	Yes	16 (17%)	12 (13%)	21 (23%)		

Marital status	Divorced	21 (23%)	20 (22%)	4 (4%)	Fisher's exact	<0.001
	Married	51 (55%)	57 (61%)	53 (57%)		
	Single	20 (22%)	16 (17%)	28 (30%)		
	Widowed	0 (0%)	0 (0%)	8 (9%)		

Highest level of education	Never studied	11 (12%)	5 (5%)	32 (34%)	Fisher's exact	<0.001
	Preprimary	6 (6%)	3 (3%)	51 (55%)		
	Primary school	42 (45%)	48 (52%)	10 (11%)		
	Secondary school	30 (32%)	23 (25%)	0 (0%)		
	Tertiary institution	2 (2%)	6 (7%)	0 (0%)		
	University	2 (2%)	7 (8%)	0 (0%)		

HIV status	Negative	52 (56%)	42 (45%)	22 (24%)	Fisher's exact	<0.001
	Positive	41 (44%)	50 (54%)	71 (76%)		
	Unknown	0 (0%)	1 (1%)	0 (0%)		

Smoking	No	92 (99%)	88 (95%)	93 (100%)	Fisher's exact	0.027
	Yes	1 (1%)	5 (6%)	0 (0%)		

Presenting complaint	Vaginal discharge	12 (12%)	7 (7%)	14 (15%)	Fisher's exact	<0.001
	Back pain	2 (2%)	3 (3%)	0 (0%)		
	Cervicitis	62 (67%)	67 (72%)	55 (59%)		
	Candidiasis	5 (6%)	2 (2%)	0 (0%)		
	Painful micturition	2 (2%)	0 (0%)	10 (11%)		
	Valvular warts	6 (7%)	8 (9%)	14 (15%)		
	Syphilis	2 (2%)	3 (3%)	0 (0%)		
	Trichomoniasis	2 (2%)	0 (0%)	0 (0%)		
	Others	0 (0%)	3 (3%)	0 (0%)		

Contraceptive use	No	59 (65%	39 (43%)	59 (63%)	Chi-square	0.009
	Yes	32 (35%)	51 (57%)	34 (37%)		

Type of contraceptive	IUD	4 (13%)	11 (21%)	17 (44%)	Fisher's exact	0.007
	Hormonal	26 (81%)	39 (74%)	22 (56%)		
	BTL	2 (6%)	3 (6%)	0 (0%)		

*Note*: Age is a continuous variable and was presented as mean (standard deviation).

Abbreviations: BP, blood pressure; BTL, bilateral tubal ligation; CC, cervical cancer; CIN, cervical intraepithelial neoplasia; IUD, intra uterine device.

**Table 2:** Individual and combined diagnostic accuracy of serum P16ink4A and FOXP3 concentrations for the detection of cervical intraepithelial neoplasia and cervical cancer from healthy controls.

Biomarker	CIN		CC	
	Percentage	95% CI	Percentage	95% CI
FOXP3				
Sensitivity	32.22%	25.40%–39.05%	57.78%	50.56%–64.99%
Specificity	68.89%	62.13%–75.65%	68.89%	62.13%–75.65%
P16ink4A				
Sensitivity	50.00%	42.70%–57.30%	60.00%	52.84%–67.16%
Specificity	56.67%	49.43%–63.91%	55.56%	48.30%–62.81%
Combination Of FOXP3 and P16ink4A				
Sensitivity	18.89%	13.17%–24.61%	33.33%	26.45%–40.22%
Specificity	83.33%	77.89%–88.78%	83.33%	77.89%–88.78%

Abbreviations: CC, cervical cancer; CI, confidence interval; CIN, cervical intraepithelial neoplasia.

## Data Availability

The data that support the findings of this study are available on request from the corresponding author. The data are not publicly available due to privacy or ethical restrictions.

## Nomenclature

AOR: Adjusted odds ratio
COR: Crude odds ratio
CI: Confidence interval
CIN: Cervical intraepithelial neoplasia
HIV: Human Immunodeficiency Virus
HPV: Human Papilloma Virus
HR: HPV High Risk Human Papilloma Virus
LEEP: Loop electrosurgical excision procedure
OR: Odds ratio
PAP: Papanicolaou
SD: Standard deviation
STATA: Statistical software for data science
VIA: Visual inspection with acetic acid
